# Core–Sheath Nanofibers From a Modified Coaxial Electrospinning for Transdermal Delivery of Finasteride

**DOI:** 10.1002/open.202500564

**Published:** 2026-06-14

**Authors:** Liang Sun, Yisa Zhao, Xiyu Caomeng, Deng‐Guang Yu, Ping Liu

**Affiliations:** ^1^ Department of Urology Shanghai General Hospital Shanghai Jiao Tong University Shanghai China; ^2^ Department of Urology Shandong Provincial Hospital Affiliated to Shandong First Medical University Jinan China; ^3^ School of Materials and Chemistry University of Shanghai for Science and Technology Shanghai China; ^4^ School of Information Science and Technology Sanda University Shanghai China; ^5^ Department of Science and Education Dongying People’s Hospital (Dongying Hospital of Shandong Provincial Hospital Group) Dongying China

**Keywords:** core–sheath nanostructures, electrospinning, finasteride, sustained release, transdermal drug delivery

## Abstract

Traditional blending electrospinning has a series of issues during implementation, and the corresponding monolithic nanofibers can always be updated through the intentional design of core–sheath nanostructures. In this study, a modified coaxial electrospinning approach was employed to refine the electrospinning process by eliminating abnormal phenomena, while simultaneously imparting core–sheath nanostructures and improved transdermal finasteride (FIN) delivery performance. Using an electrospinnable ethylcellulose (EC) solution containing FIN as the core fluid and an unspinnable polyvinylpyrrolidone (PVP)/sodium dodecyl sulfate (SDS) solution as the sheath fluid, medicated core–sheath nanofibers (E3) were fabricated continuously and robustly. The monolithic nanofibers (E2) from a conventional blending electrospinning were prepared as a comparison. FIN existed in an amorphous state within both E2 and E3 nanofibers, attributed to its excellent compatibility with EC. The sustained release profiles of nanofibers E2 and E3 are similar, but the penetration rates of FIN molecules from the nanofibers E2 and E3 always have significant differences from the initial tests at 0.5 h (10.32 ± 4.31% and 12.85 ± 5.14% for nanofibers E2 and E3, respectively) to the final tests at 4.0 h (38.16 ± 5.47% and 51.31 ± 5.19% for nanofibers E2 and E3, respectively). The mechanism underlying the robust preparation enabled by the unspinnable sheath fluid is proposed: it prevents multiple abnormal phenomena inherent to single‐fluid blending processes. This protocol offers a versatile strategy to upgrade traditional single‐fluid blending electrospinning products, endowing them with an improved functional performance.

## Graphical abstract

A modified coaxial electrospinning was conducted to prepare core–sheath nanofibers. They have transdermal enhancer sodium dodecyl sulfate (SDS) and soluble polyvinylpyrrolidone in their sheaths, and finasteride and insoluble ethylcellulose in the cores. The fast release of SDS and sustained release of finasteride can jointly provide an improved transdermal effect, showing potential applications in treating prostatitis.

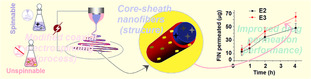

## 1 Introduction

Transdermal delivery is an intriguing route of drug administration that involves applying a formulation onto intact skin [1, 2, 3, 4, 5, 6, 7]. The underlying mechanism is the penetration of the drug across the stratum corneum and through skin appendages into subcutaneous capillaries [3, 8, 9, 10, 11, 12]. By bypassing gastrointestinal degradation and the hepatic first‐pass effect, this noninvasive technology offers prolonged, steady systemic exposure [13, 14]. It is already used to manage moderate‐to‐severe cancer pain, deliver anesthetic agents and veterinary anthelmintics, and treat cardiovascular disease [15, 16]. According to industry estimates, the global transdermal patch market will reach USD 9.6 billion by 2027 [17], and the global burden of the diseases that transdermal delivery could lead to positive effects was estimated to be around USD 31.6 billion even in 2015, and is always increasing [18].

Just as with other drug delivery routes, the corresponding systems must be updated with new pharmaceutical excipients and modern materials‐conversion techniques to incorporate both the drug and its carriers into well‐defined dosage forms [19, 20, 21, 22, 23, 24]. Over the past two decades, electrospinning and the resulting nanofibers have attracted increasing attention for a range of medical devices in which drug delivery is the primary function [25, 26, 27, 28, 29]. Electrospun nanofibrous wound dressings capable of providing predetermined release profiles of single or multiple (even three) active ingredients have been reported extensively [30, 31, 32, 33, 34]. Nevertheless, electrospun medicated nanofibrous films designed specifically for transdermal drug delivery remain scarce. Moreover, such films have almost exclusively been fabricated by the traditional single‐fluid blending electrospinning process [35, 36], although the field is rapidly evolving toward multi‐fluid strategies [37, 38]. These newer strategies encompass bi‐fluid coaxial [39, 40, 41] and side‐by‐side [42] configurations, as well as tri‐fluid triaxial (tri‐fluid coaxial), tri‐fluid side‐by‐side, and hybrid arrangements that combine coaxial and side‐by‐side geometries. They enable the creation of diverse complex nanostructures—core–sheath, Janus, trichamber core–sheath, trichamber Janus, and trichamber hybrids—that can serve as robust platforms for next‐generation drug‐delivery devices, including novel transdermal films [43, 44, 45, 46, 47, 48, 49, 50, 51]. We therefore hypothesize that core–sheath nanofiber mats fabricated by a modified coaxial electrospinning process could allow transdermal enhancers and active ingredients to be released asynchronously, thereby improving transdermal efficiency.

Finasteride (FIN) is a medication used to treat and control benign prostatic hyperplasia (BPH). It reduces the risk of acute urinary retention and the need for transurethral prostatectomy or prostatectomy [52, 53]. FIN shrinks an enlarged prostate, improves urine flow, and alleviates BPH‐related symptoms, making it suitable for patients with prostate enlargement [54]. However, oral FIN tablets may cause side effects, including breast hyperplasia and pain, decreased libido, erectile dysfunction, reduced ejaculation volume, allergic skin reactions (rash, itching, urticaria, lip swelling), and liver damage (abnormal liver function, hepatitis) [55]. Therefore, FIN is selected here as a model drug for electrospun transdermal nanofibrous films.

**SCHEME 1:**
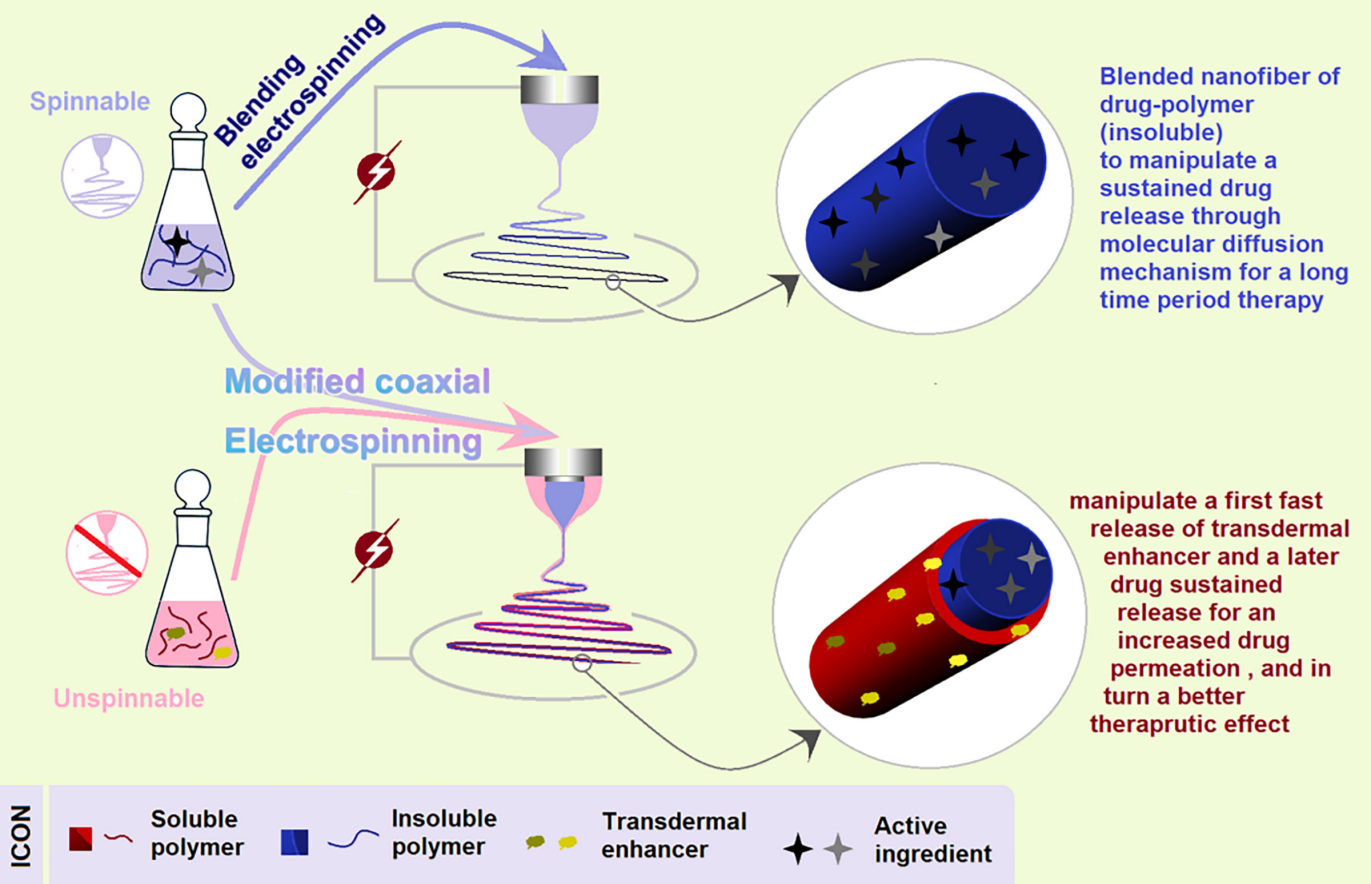
A direct comparison between the blending electrospinning and the modified coaxial electrospinning and their corresponding nanofibers.

In the pharmaceutical field, soluble and insoluble polymeric excipients are frequently used to manipulate the release behavior of loaded drugs [56]. Here, polyvinylpyrrolidone (PVP) and ethylcellulose (EC) were selected as the sheath and core matrices, respectively, to enable rapid release of the transdermal enhancer and sustained release of FIN from electrospun core–sheath nanofibers. Sodium dodecyl sulfate (SDS), a widely used anionic surfactant in pharmaceuticals, plays a key role in drug formulation development, medical device disinfection, and diagnostic reagents due to its strong emulsifying, solubilizing, and bactericidal properties [57]. The transdermal mechanism of SDS is primarily governed by its molecular structure and interactions with the skin surface. As an anionic surfactant, its hydrophobic alkyl chain (C_12_H_25_) adsorbs onto skin lipids or the stratum corneum, while the hydrophilic sulfonate group (SO_4_Na) extends into the aqueous phase, forming a surface‐active layer. This configuration reduces surface tension and promotes the spreading and penetration of drug molecules on the skin [58]. For comparison, homogeneous FIN‐loaded EC nanofibers were also prepared via single‐fluid blended electrospinning. A direct comparison between them is shown in Scheme 1.

## 2 Materials and Methods

### 2.1 Materials

EC (viscosity 6–9 mPa·s) was purchased from Aladdin Chemistry Co., Ltd. (Shanghai, China). FIN, PVP K10 (*M*_W_ = 8000), SDS, dichloromethane (DCM), dimethylacetamide (DMAc), basic fuchsin, and anhydrous ethanol were obtained from Sinopharm Co., Ltd. (Shanghai, China). Phosphate buffer solution (PBS, 0.1 M, pH 7.0) was purchased from Tianjin Zhiyuan Chemical Reagent Co., Ltd. (Tianjin, China). Ultrapure water was freshly double‐distilled before use. All reagents were used as received.

### 2.2 Electrospinning

A laboratory‐scale electrospinning system was constructed to prepare nanofibers; its key feature was a custom‐designed concentric spinneret. The setup comprised two syringe pumps (one KDS‐100 and one KDS‐200, Cole‐Parmer, USA) for precise delivery of the working fluids, a high‐voltage power supply (ZGF 60 kV/2 mA, Wuhan Hua‐Tian CO., Ltd., China) to generate the required electric field, and a grounded collector—fabricated for safety by wrapping aluminum foil around rigid cardboard. Two working fluids, whose compositions and operating parameters are listed in Table 1, were selected after preliminary trials. Process visualization was achieved with a Canon G7X digital camera (Canon, Japan) at various magnifications.

**TABLE 1:** The parameters of three EHDA processes.

No.	Process	Fluid/Flow rate, mL/h		Operational parameters
		Sheath^a^	Core^b^	
E1	Blending electrospraying	Fluid 1/2.0	—	Applied voltage: 12 kV Collection distance: 20 cm Environmental Temp: 21 ± 3°C Relative humidity: 51 ± 6%
E2	Blending electrospinning	—	Fluid 2/2.0	
E3	Modified coaxial electrospinning	Fluid 1/1.0	Fluid 2/1.0	

a Shell fluid PVP solution consisted of 20.0 g PVP K10 and 3.0 g SDS in 100 mL ethanol/DMAc mixture (80:20 in volume).

b Core fluid EC solution consisted of 22.0 g EC and 3.0 g FIN in 100 mL ethanol/DCM mixture (50:50 in volume).

### 2.3 Characterizations of Physical Properties

#### 2.3.1 Morphology

Surface morphology of the three EHDA products was evaluated using scanning electron microscopy (SEM; Quanta 450 FEG, FEI, Hillsboro, OR, USA). Before imaging, specimens were sputter‐coated with platinum for 60s to ensure adequate conductivity. The applied voltage for gathering the SEM images was fixed at 20 kV. Fiber diameters and size distributions were subsequently analyzed from the acquired micrographs using ImageJ software (National Institutes of Health, Bethesda, MD, USA).

#### 2.3.2 Inner Structure

Transmission electron microscopy (TEM, JEOL, Tokyo, Japan) was employed to probe the internal architecture of the three EHDA products. To prepare specimens, a carbon‐coated copper grid was positioned directly beneath the spinneret and immediately above the collector to collect the samples for approximately 30 s. The voltage applied to the samples during the TEM observations was fixed at 300 kV.

#### 2.3.3 Physical State

A Bruker D8 Advance X‐ray diffractometer (Bruker, Germany) was used to assess the physical form of the raw FIN, EC, PVP, and SDS powders, as well as the physical state of FIN within the three EHDA products (E1, E2, and E3). The instrument used a Cu Kα X‐ray source operated at 40 kV and 40 mA. Data were collected from 5° to 60° 2θ with a step size of 0.02°and a scan rate of 10°/min^−1^.

#### 2.3.4 Compatibility

Attenuated Total Reflection‐Fourier transform infrared (ATR‐FTIR) spectra of neat FIN, EC, SDS, and PVP powders and their EHDA products were measured using a PerkinElmer spectrometer (Billerica, MA, USA). Measurements were performed over 500–4000 cm^−1^ at a spectral resolution of 2 cm^−1^.

#### 2.3.5 Tensile Strength Test

A Precisionline Vario Tensile Testing Machine (Zwick Co., Ltd., Germany) was exploited to evaluate the tensile strength (TS) and elongation at break (EB) of the nanofibrous membranes E2 and E3. The tests were conducted at a tensile speed of 1.0 mm/min. The thickness of the nanofibrous membranes was measured using a micrometer.

#### 2.3.6 Water Contact Angle

The water contact angle (WCA) of the nanofibrous membranes E2 and E3 was determined using a contact angle analyzer (JC2000C1, Shanghai Zhongchen Digital Technology Equipment Co., Ltd., Shanghai, China). The images were subjected to processing and analysis through ImageJ software to examine the dynamic behavior of water droplets on the fiber membrane. The experiments were repeated six times.

### 2.4 Characterizations of Functional Performances

#### 2.4.1 Quantitative Measurements of FIN

A UV–Vis spectrophotometer (model UV‐2102C, Unico Instrument Co. Ltd., Shanghai, China) was used for all quantitative analyses of FIN. A calibration curve was first constructed with standard solutions to relate absorbance to concentration.

#### 2.4.2 Drug Encapsulation Efficiency (EE%) and Drug Loading (DL%)

FIN was recovered from the nanofibers E2 and E3. A certain weight of fibrous films (W_f_) was dissolved in anhydrous ethanol. A volume of 1.0 mL of the ethanolic solution was gradually added to 1.0 L distill water under stirring to liberate FIN. After centrifugation (5000 rpm, 10 min), the supernatant was measured. The drug mass in the fibrous film (W_d_) can be calculated. Thus, DL% was computed as Equation (1):

(1) $$\text{DL}\%=(W_{d}/W_{f})\times 100$$

And the EE% can be calculated according to Equation (2)

(2) $$\text{EE}\%=(W_{d}/W_{p})\times 100$$

where *W*_p_ is the mass of FIN initially added to the working fluid for preparations.

#### 2.4.3 *In* *V* *itro* Release Studies

The release kinetics of FIN from electrospun nanofibers (formulations E2 and E3) were evaluated by the paddle method specified in the Chinese Pharmacopeia (2020 Edition) as follows: 1) Nanofiber samples equivalent to 20 mg FIN were placed in vessels containing 600 mL PBS (pH 7.4), thermostated at 37°C. The paddles rotated at 50 rpm. 2) At predetermined intervals, 5.0 mL aliquots were withdrawn and immediately replaced with an equal volume of fresh PBS to maintain sink conditions. 3) The absorbance of each sample was measured with the UV‐Vis spectrophotometer and converted to concentration (C, μg mL^−1^) using the calibration curve. Cumulative release (%) was calculated with Equation (3):

(3) $$P(\%)=\frac{C_{n}\times V_{0}+\sum_{i=1}^{n-1}C_{i}\times V}{Q_{0}}\times 100$$

where *V*_0 _ is the total dissolution medium volume (600 mL), *V* is the  sample volume removed (5.0 mL), *Q*_0_ is the absolute FIN content in the nanofibers (mg), *C*_*n*_ is the concentration in the nth sample (mg L^−1^), and *C* *_i_* is the concentration in the *i* ^th^ sample (mg L^−1^).

#### 2.4.4 Ex Vivo Permeation Investigations

Ex vivo permeation studies were conducted using an RYJ‐6A diffusion apparatus (Shanghai Huanghai Drug Control Instrument, China) equipped with six Keshary‐Chien glass diffusion cells. A water bath maintained the receptor medium at 37 ± 0.5°C throughout the experiment. Each cell exposed 2.60 cm^2^of membrane surface area and contained 7.2 mL of receptor fluid. Fresh porcine belly skin, collected from a local abattoir within 2 h of slaughter, was carefully excised and mounted between the donor and receptor compartments with the epithelial side facing upward. The donor compartment was loaded with 1.0 mL of PBS. A magnetic bead stirred the receptor medium at 100 rpm to ensure uniform hydrodynamics. After a 30‐min equilibration period, a certain amount of E2 and E3 (containing 125 µg FIN) was applied. At predetermined intervals, 1.0 mL aliquots were withdrawn from the receptor compartment, immediately filtered through 0.22 µm membranes (EMD Millipore), and analyzed spectrophotometrically at 220 nm for FIN content. Each experiment was performed in sextuplicate (*n*  =  6).

### 2.5 Statistical Analysis

All data are reported as mean ± standard deviation (SD). All the quantitative measurements were repeated at least three times (n ≥ 3). One‐way analysis of variance (ANOVA) was employed to detect significant differences among groups. When ANOVA indicated significance (*p* < 0.05), the Dunnett’s post‐hoc test was applied to identify specific differences.

## 3 Results and Discussion

### 3.1 Modified coaxial electrospinning based on a combination of electrospraying and electrospinning

**FIGURE 1:**
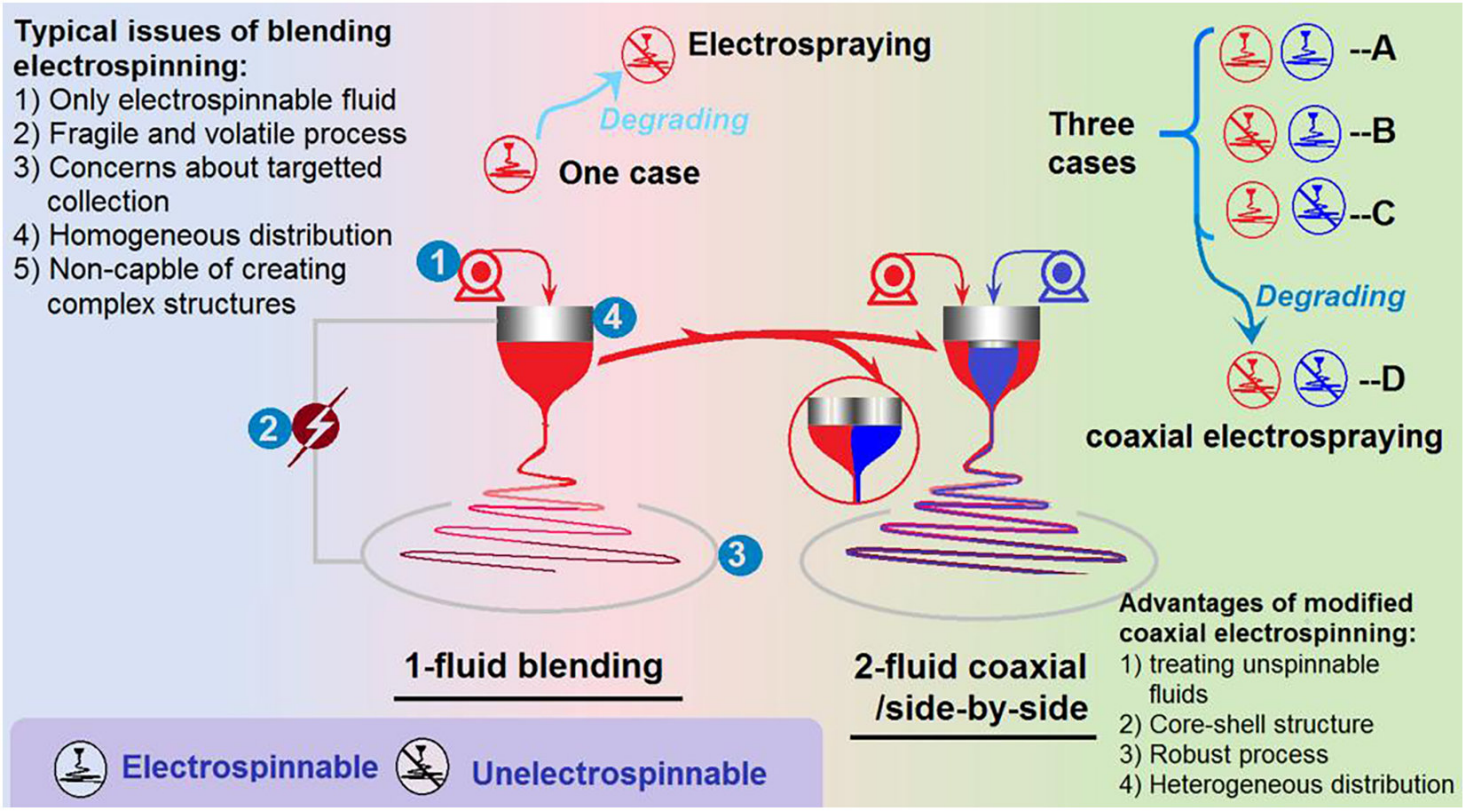
A diagram about the components of an EHDA apparatus (1–pump, 2–power supply, 3–collector, and 4–spineret), the typical issues of the single‐fluid blending electrospinning, and the advantages of the developed modified coaxial electrospinning.

Both electrospraying and electrospinning belong to the electrohydrodynamic atomization (EHDA) methods [59]. The apparatuses are similarly composed of at least four components: 1–pump, 2–power supply, 3–collector, and 4–spineret, as indicated in Figure 1. The most fundamental electrospinning process is the single‐fluid blending electrospinning, which was revived in the 1990s when nanoscience began to soar [60]. However, there are a series of typical issues during the implementations of the single‐fluid blending processes and its products, which include (1) the treated working fluids must be electrospinnable, or the process would be degraded into an electrospraying process; (2) the working processes are often fragile and volatile, very sensitive to the environmental conditions; (3) all the blended components within the electrospun nanofibers are homogeneously distributed; (4) the process is incapable of creating complex nanostructures; and (5) concerns about satellite droplets or nozzle clogging are frequently raised.

For overcoming the limitations of the single‐fluid blending electrospinning, a series of multiple‐fluid processes has been successively developed. These processes have at least two aspects to expand the capability of electrospinning in creating nanofibers. One is the co‐treatment of one or even several unspinnable fluids. During the multi‐fluid electrospinning processes, often only one of the working fluids must be electrospinnable; other fluids can be electrospinnable or can be unspinnable. As indicated in Figure 1, there are four types of combinations of the core fluid and sheath fluid, that is, both of them are electrospinnable (as indicated by A), an electrospinnable sheath and an unspinnable core (case C). These two cases belong to the traditional coaxial electrospinning. Case B has an unspinnable sheath but an electrospinnable core, representing a typical modified coaxial electrospinning, the working process developed here. As for case D, it is a coaxial electrospraying process because both the sheath and core fluids are unspinnable.

The modified coaxial electrospinning can be viewed as a combination of the single‐fluid blending electrospinning of the core liquid and the single‐fluid blending electrospraying. The additional sheath unspinnable can be managed to endow the working processes and also the corresponding nano products with several advantages. One advantage is that numerous unspinnable liquids can be converted into nanofibers with a core support to take advantage of the unique properties of electrospun nanofibrous mats. Meanwhile, the designed core–sheath functional nanostructures can be greatly expanded because the electrospinnable solutions are very limited, but the unspinnable fluids are numerous. Certainly, based on the core–sheath nanostructures, the heterogeneous distributions of active ingredients in the nanoscale can be facilely realized, such as gradient distributions of drug molecules [61], gradient distributions of polymeric matrices [62], and surface distribution of pure drug molecules [63]. Additionally, the sheath unspinnable fluid can be exploited to endow a more continuous and robust preparation process by eliminating a series of abnormal phenomena associated with the high viscosity of the electrospinnable liquids.

### 3.2 Implementations of the three types of modified coaxial electrospinning

The successful implementation of a coaxial electrospinning has two important elements. One is the reasonable design of the concentric spinneret, which is the most important and innovative part within an electrospinning apparatus. The other is the compatibility between the sheath and core working fluids; at least coagulation and gelling should be avoided between them.

**FIGURE 2:**
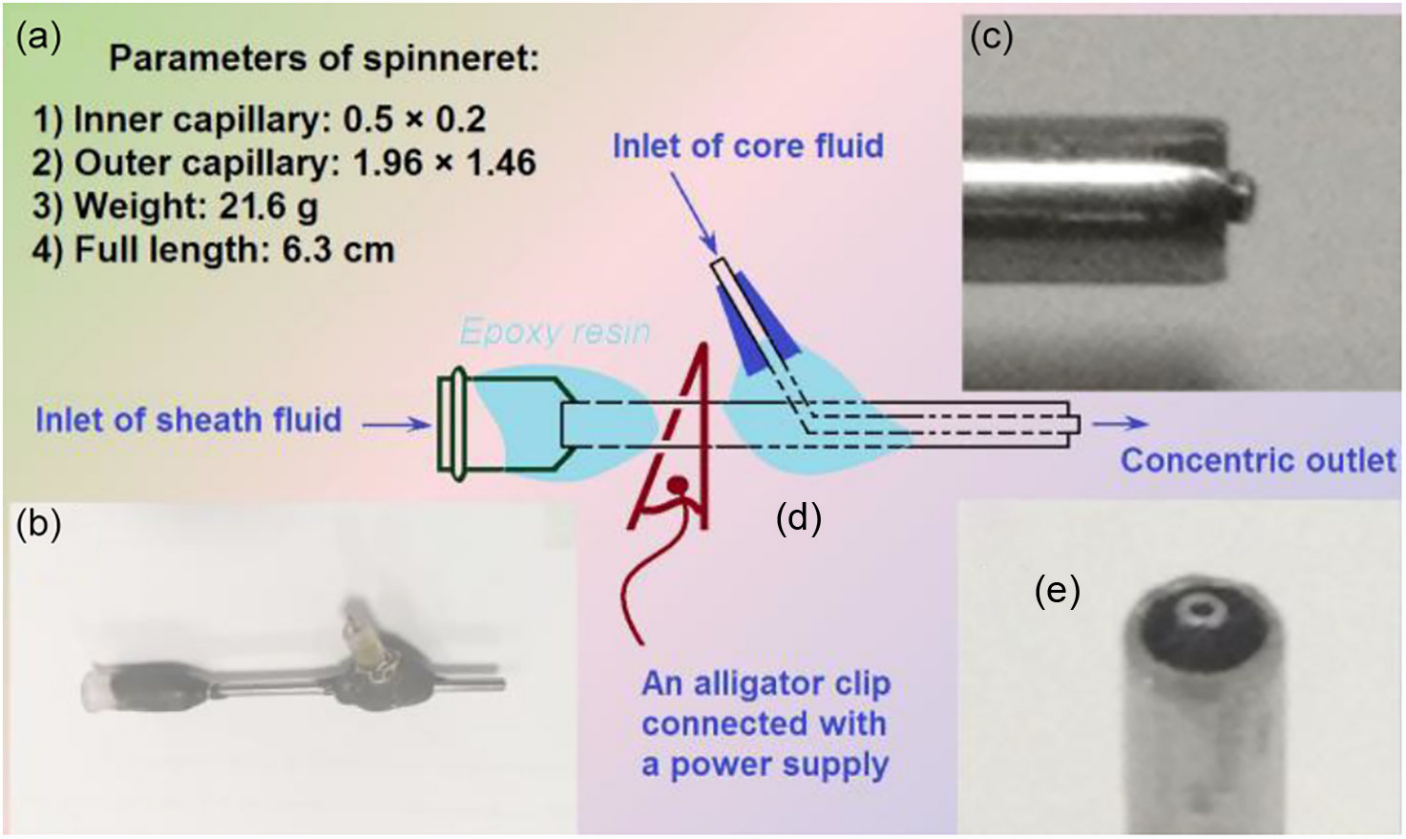
The specially designed spinneret structure: (a) a traditional side‐by‐side spinneret composed of two parallel metal capillaries and a section PP tube with varying diameters; (b) a simple insertion of the PP tube onto the metal capillaries can form the core‐shell fluid‐guiding exit; (c) a digital photo of the tri‐fluid coaxial nozzle of the structural spinneret; (d) the connections of the spinneret with three syringes containing the three different working fluids. A sharp needle is used to penetrate the PP tube to directly transfer the outer core‐shell fluid, as indicated by the red arrow; (e) a schematic diagram showing the inner structure characteristics of the spinneret.

In this study, the designed concentric spinneret is shown in Figure 2. The spinneret has mainly two stainless steel capillaries. One tube has inner and outer diameters of 1.46 mm and 1.96 mm, respectively, and it was exploited as the sheath tube. The other tube has inner and outer diameters of 0.2 mm and 0.5 mm, respectively. It was nested into the larger capillary in a concentric manner to act as the core capillary. Epoxy resin was used to fix all the parts due to its advantage of having almost no volume change during the solidification process, and it is therefore popular as an adhesive in many fields. The device has a total length of 6.3 cm and a total weight of 21.6 g. It is very light for fixing and operation, and it is safer owing to its effective prevention of the spinneret separating from the syringe in a vertical electrospinning apparatus.

The inner structural characteristics of the concentric spinneret are exhibited in Figure 2a, with two separate inlets for guiding the core and sheath liquids to the only concentric outlet. A digital image of the concentric spinneret is given in Figure 2b. Horizontal and vertical views of the outlet are shown in Figure 2c,d, respectively. The inner capillary slightly projects out of the outer capillary 0.5 mm, which can be exploited for a better encapsulation of the inner fluid by the outer fluid and also for preventing the possible diffusion of the components in the core and sheath fluids.

**FIGURE 3:**
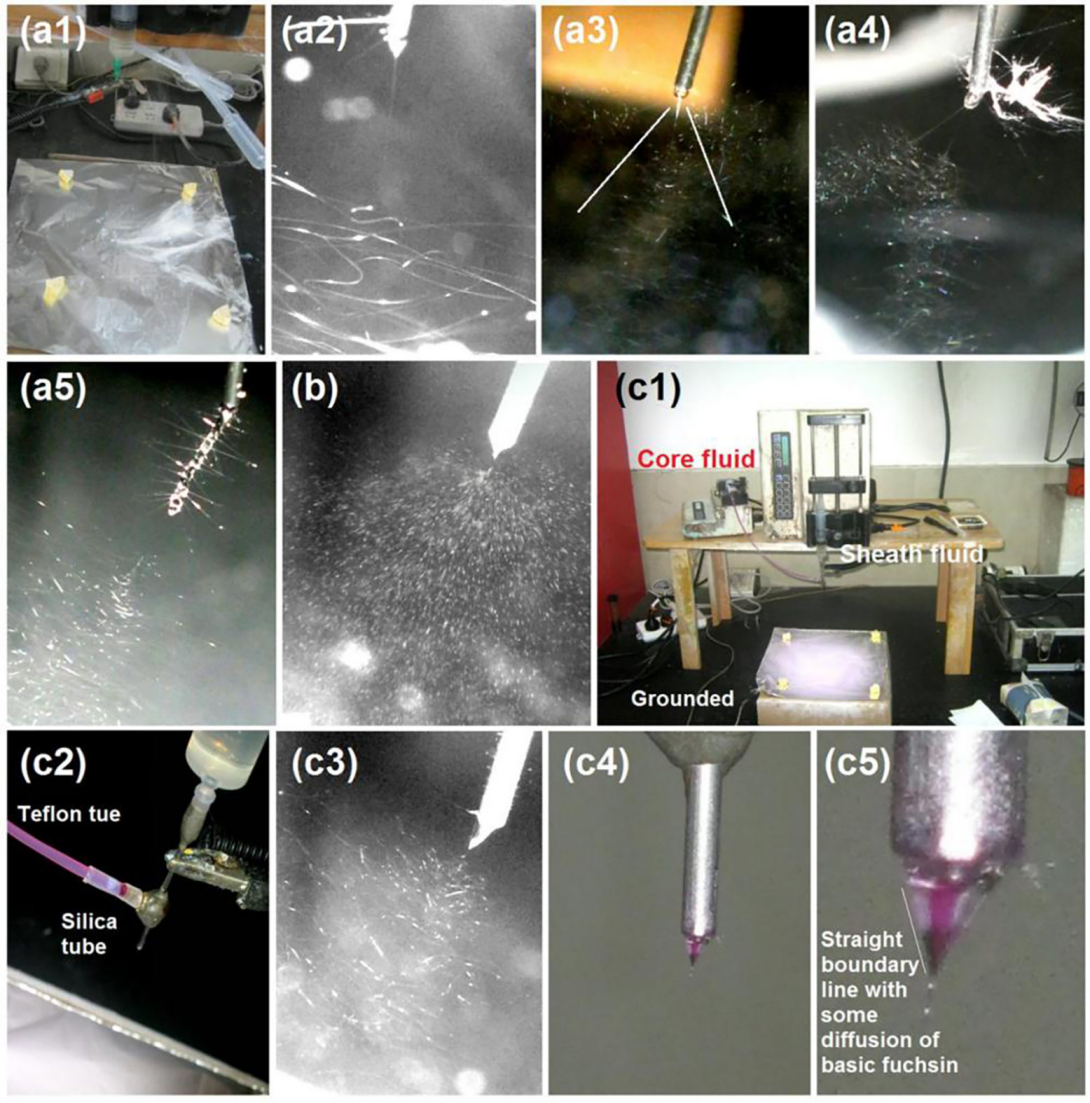
The implementations of the three EHDA processes: (a1) the apparatus of the single‐fluid blending electrospinning of the core fluid; (a2) the typical electrospinning processes of the core fluid; (a3) the fluid jet was divided into three branches due to a higher applied voltage; (a4) the splittings of fluid jet and meanwhile the hanging of semi‐solid substance; (a5) the continuous electrospinning from the formed semi‐solid substance hung on the nozzle of spinneret; (b) a typical electrospraying process for creating microparticles E1; (c1) a bird’s‐eye view of the homemade electrospinning apparatus for the modified coaxial electrospinning; (c2) the connection of spinneret with two syringe pumps and the power supply through an alligator clip; (c3) a typical coaxial working process; (c4) the core–sheath Taylor cone; (c5) an enlarged image of the core–sheath Taylor cone.

The photos of the implementations of the three EHDA processes and some abnormal phenomena are exhibited in Figure 3. The apparatus of the single‐fluid blending electrospinning of the core fluid is shown in Figure 3a1. A monoaxial metal capillary was exploited to create the medicated nanofibers F2. The typical electrospinning processes of the single EC and FIN co‐dissolved working fluid are shown in Figure 3a2, in which the well‐known three successive steps, that is, Taylor cone, straight fluid jet, and a bending and whipping region with enlarged circles are obvious. However, some abnormal phenomena appeared now and then, and manual involvement was needed for continuous preparation. As shown in Figure 3a3–a5 are three typical cases. As shown in Figure 3a3, the fluid jet was easily divided into several branches when a slightly higher applied voltage of 14 was applied, as separated by two white lines. In Figure 3a4, not only did the divisions of fluid jets appear but also the hanging of semi‐solid substance on the nozzle of the spinneret was clear. The formation of semi‐solid substances could bring out other abnormal situations. As indicated in Figure 3a5, the electrospinning process could still be moved forward from the tip of the semi‐solid column. The core section of the semi‐solid column was still in a liquid state. The sheath fluid composed of PVP K10 and SDS was unspinnable due to no enough physical entanglements from the relatively small molecules of PVP K10 to resist the electrical drawing. A typical electrospraying process was exhibited in Figure 3b. As anticipated and similar to the single‐fluid electrospinning, there were three successive steps from the formation of the Taylor cone, the convergent point, and the Coulombic explosions in the unstable atomization region.

The single‐fluid electrospraying process and the double‐fluid electrospraying process can be integrated into a modified coaxial electrospinning process using a similar apparatus. As indicated in Figure 3c1, it is a bird’s‐eye view of the homemade electrospinning apparatus for the modified coaxial electrospinning. Similarly, there were four parts, that is, pump, the power supply, the collector, and the spinneret. The differences lie in that a concentric spinneret was exploited and two pumps were needed to quantitatively drive the core and sheath working fluids. The spinneret is the convergent point. Its connections with two syringes and the power supply through an alligator clip are indicated in Figure 3c2. A silica tube could absorb DCM. Thus, the Teflon tube was exploited to guide the EC‐FIN fluid with a silica tube as a connection due to its high elasticity. Under the experimental conditions, the typical coaxial electrospinning process was shown in Figure 3c3, in which the Taylor cone, straight fluid jet, and the gradual enlargements of bending and whipping circles were obvious. The compound core–sheath Taylor cones were exhibited in Figure 3c4, in which it is clear that the core EC‐FIN fluid was encapsulated by the sheath PVP‐SDS fluid. The red color was a result of the basic fuchsin with a concentration of 10^−6^ g/mL. An enlarged image of the Taylor cone was shown in Figure 3c5, from which it is clear that the boundary line between the sheath fluid and the surrounding environment was a straight line. This is different from those boundaries of the single‐fluid electrospinning processes in Figure 3a2–3a4, which were arc lines. It is the unspinnable property of the sheath fluid that brought out the straight boundary lines. Meanwhile, there is a very slight diffusion of basic fuchsin molecules from the core section to the sheath section of the Taylor cone. It should also be the diffusion that the nanofibers E3 deposited on the collector in Figure 3c1 had a very slight red color.

### 3.3 Morphologies and inner structures of the prepared nanofibers

**FIGURE 4:**
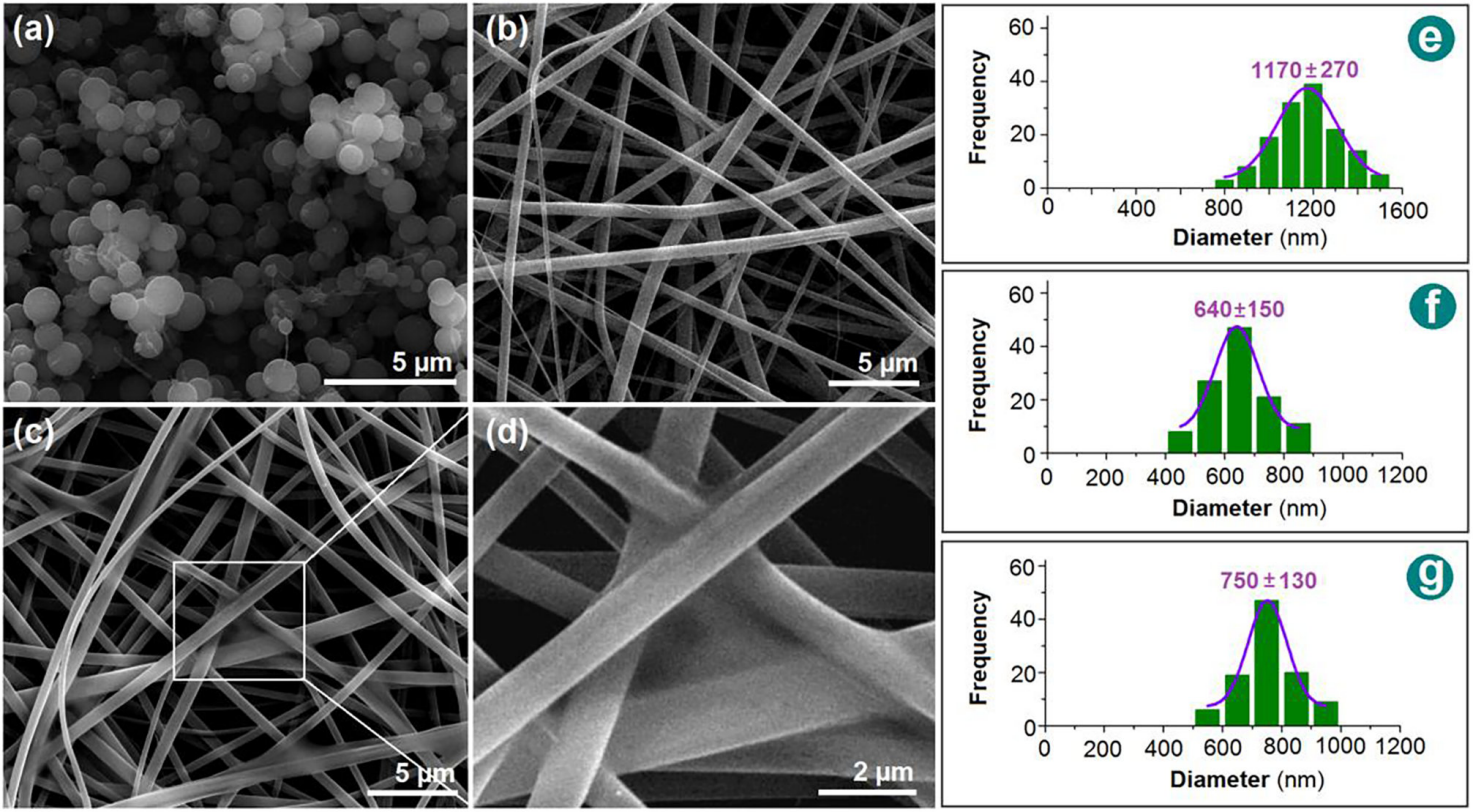
SEM images of the three kinds of EHDA products: (a) microparticles E1; (b) nanofibers E2; and (c1 and c2) nanofibers E3. Their size and size distributions, where (e), (f), and (g) are the values for E1, E2, and E3, respectively.

The SEM images of the three kinds of EHDA products, their diameters, and size distributions are shown in Figure 4. As indicated in Figure 4a, the electrosprayed PVP‐SDS composites had a round particulate morphology, which had an average diameter of 1.17 ± 0.27 μm (Figure 4e). As anticipated, nanofibers E2 had a linear morphology, whose average diameters were 640 ± 150 nm, as indicated in Figure 4b,f. Nanofibers E3 (Figure 4c) had a linear morphology similar to E2. Their diameters were 750 ± 130 nm (Figure 4g), which was slightly larger than that of monolithic nanofibers E2. An interesting phenomenon was captured in Figure 4d, in which the sheath PVP K10 was spread among the close nanofibers, which may be a result of the environmental moisture.

**FIGURE 5:**
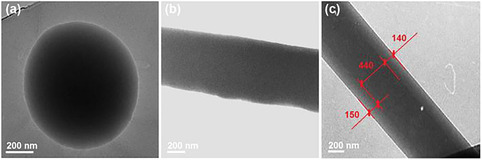
TEM images of the three kinds of EHDA products: (a) microparticles E1; (b) monolithic nanofibers E2; and (c) core–sheath nanofibers E3.

The TEM images of the three kinds of EHDA products are exhibited in Figure 5. As expected, the microparticles E1 had a gradient and smooth decrease gray level from the center of the micro particle to its boundary due to the thickness (Figure 5a). Similarly, the monolithic nanofibers E2 had an even gray level owing to the homogeneous distributions (Figure 5b). The core–sheath nanofibers E3 had a clear difference in the gray level between the core and sheath sections (Figure 5c). The thicknesses of the core and sheath sections are about 440 nm and 140 ~ 150 nm. Based on these data, the density differences of the core and sheath sections can be estimated as follows:

The core diameter is about 440 nm; thus, the surface area of the core section is (220  ×  220 × π)  =  48 400π. The nanofiber diameter is about (440 + 140 + 150)  =  730 nm; thus, the surface area of the circular ring section is [(730/2)^2 ^× π – (220  ×  220×π)] = 84 825π. Based on the experimental conditions, the weight of the core can be represented by the concentrations of solutes, that is, 22 + 3 = 25. The weight of the sheath can be represented by the concentrations of SDS and PVP, that is, 20 + 3 = 23. The raw density of PVP and EC 1.144 g/cm^3^ are 1.45 g/cm^3^, respectively. Thus, the packing density of the core to sheath section can be estimated as [(25/48 400π) ÷ (23/84 825π)] × (1.144/1.45) = 1.499.

**FIGURE 6:**
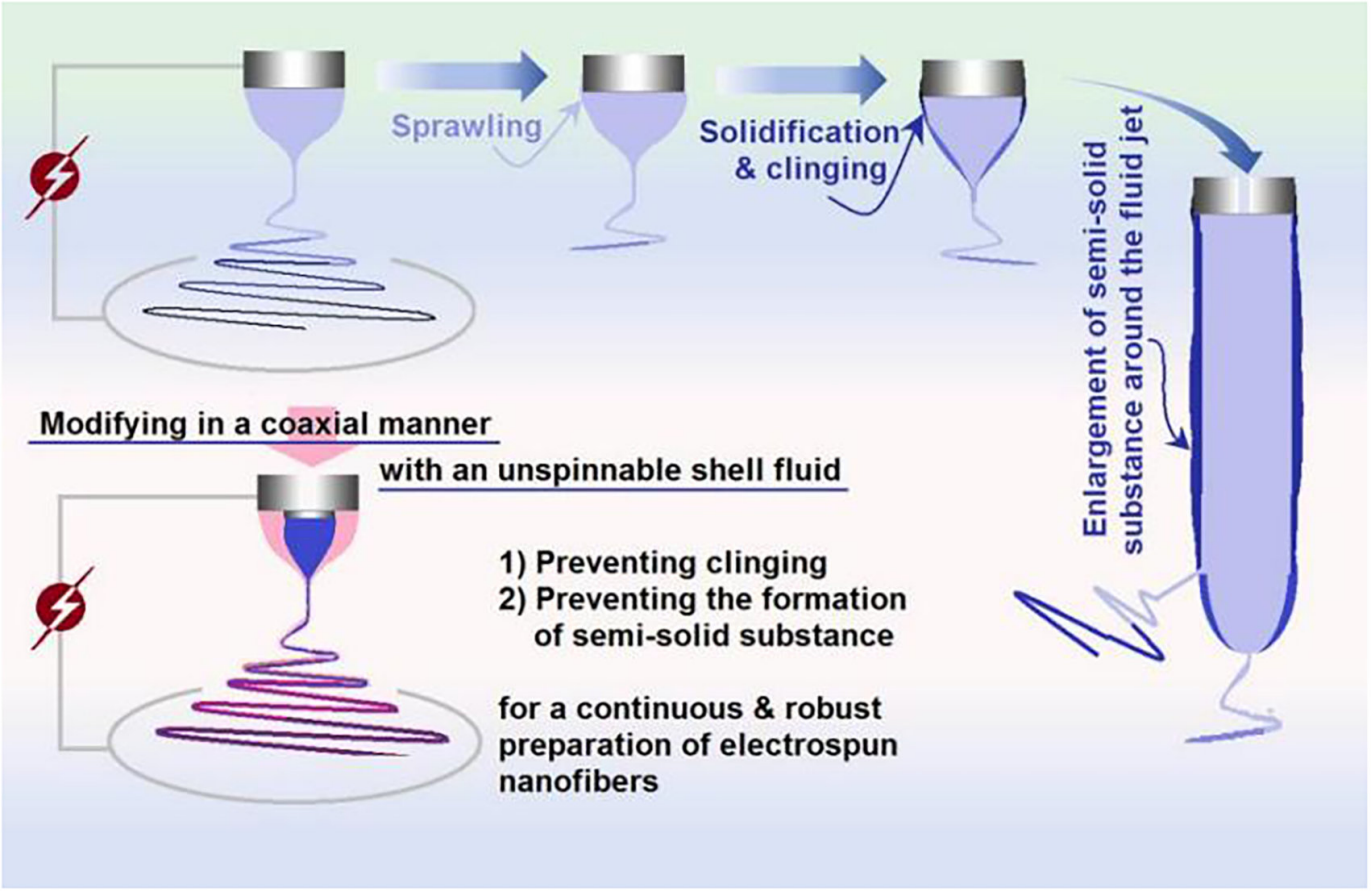
The suggested mechanisms of the influences of unspinnable sheath solution on the nano fabrications of the electrospun core–sheath nanofibers E3.

The mechanism of the clogging of the spinneret during the single‐fluid blending electrospinning is diagrammed in Figure 6. The culprit is the volatile ethanol and DCM. When the fluid is pumped out from the nozzle of the spinneret, the solution would sprawl upward on the surface of the metal capillary. Later, the fast evaporation of DCM and ethanol would convert the very viscous, sprawled fluid into a semi‐solid substance, which would cling and hang onto the nozzle. Meanwhile, the evaporation of ethanol and DCM would also form a semi‐solid substance around the fluid jet, by which the Taylor cone would be gradually enlarged. Although there is a surrounding semi‐solid substance, the core section is still the fluid jet. Thus, the electrospinning process could still go on from the enlarged Taylor cone and straight fluid jet, as indicated in Figure 3a5.

When an unspinnable shell fluid is applied, the diluted solution and small viscosity will not form a semi‐solid substance hanging and clings on the nozzle of the spinneret, eliminating also the possible formation of a semi‐solid substance around the fluid jet during the Taylor cone and straight fluid jet. In addition to ensuring a continuous and robust electrospinning process, the unspinnable shell fluid can also prevent the working process from the disturbance of the ambient conditions, and in turn endow the preparation of high‐quality nanofibers.

### 3.4 Physical state and compatibility of the components in the electrospun nanofibers

**FIGURE 7:**
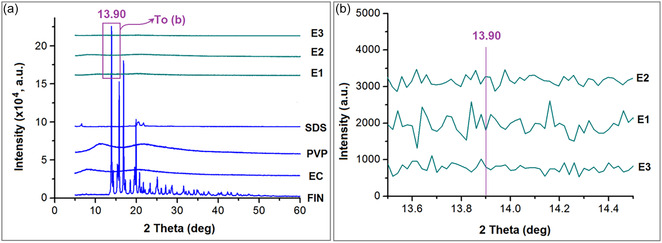
XRD patterns of the created microparticles E1, the medicated nanofibers E2 and E3, and the powders of the raw materials (FIN, SDS, PVP, and EC): (a) a same *y*‐axis scale range of all the tested samples; (b) a reduced *y*‐axis scale range and also a reduced *x*‐axis scale range to enlarge the signals at the place of 13.90°.

It is frequently reported that electrospinning is useful for transferring the poorly water‐soluble drugs from the original crystal state to a polymer‐based amorphous state, which is favorable for the dissolution and controlled release of drug molecules from the polymeric matrices [64, 65, 66, 67]. XRD patterns of the created medicated microparticles E1, nanofibers E2 and E3, and the powders of the raw materials (FIN, SDS, PVP, and EC) are exhibited in Figure 7a. The sharp peaks in the patterns of FIN and SDS indicate that the raw SDS and FIN particles are crystalline materials. The halos EC and PVP suggest that they are amorphous polymers. The patterns of the microparticles E1, the monolithic nanofibers E2, and core–sheath nanofibers E3 are highly similar, which have been reported for many other polymeric composites [68, 69, 70, 71]. They show almost straight lines of their patterns without any discernible sharp Bragg peaks, giving hints that these composite materials are presented in an amorphous state. To further demonstrate the amorphous state, the signals at the place of 13.90°, that is, the sharpest peak in the FIN patterns, are magnified through the reductions of *x*‐axis and *y*‐axis scale ranges. The results are shown in Figure 7b. It is easy to understand that there is no sharp peak signal on the patterns of microparticles E1 due to no FIN in the electrosprayed fluid. The similar, no sharp peak signal of the patterns of nanofibers E2 and E3 should be attributed to the amorphous state of FIN within the EC matrix. The inert property of EC, its hydrophobic carbon chain, and the low solubility of FIN would be favorable factors for the formation and long‐time preservation of the amorphous fibrous nanocomposites [72, 73]. The extremely fast drying processes of electrospinning and coaxial electrospinning would propagate the homogeneous state of active ingredients and polymeric carrier (here are FIN and EC) in the working fluid to the solid nanofibers [74, 75]. Thus, both E2 and E3 should be solid dispersion at a molecular scale. Meanwhile, EC molecules can act as a spatial hindrance to prevent the aggregation of FIN molecules and the formation of a crystal nucleus.

**FIGURE 8:**
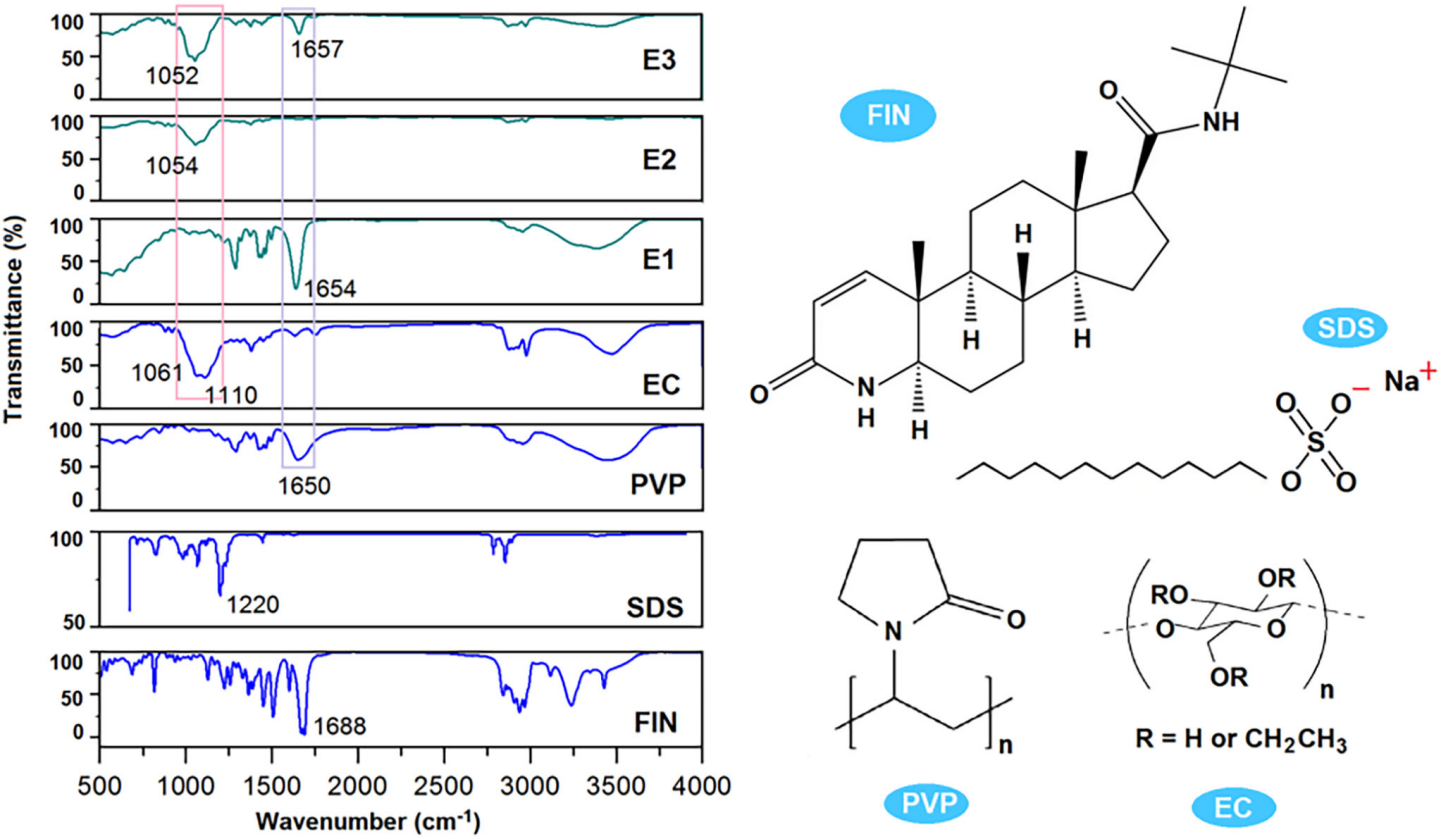
ATR‐FTIR spectra of the created microparticles E1, the medicated nanofibers E2 and E3, and the powders of the raw materials (FIN, SDS, PVP, and EC) and the formula of FIN, PVP, SDS, and EC molecules.

The ATR‐FTIR spectra of the created microparticles E1, the medicated nanofibers E2 and E3, and the powders of the raw materials (FIN, SDS, PVP, and EC) are exhibited in Figure 8. The comparisons between raw FIN with nanofibers E2 and nanofibers E3, between raw EC with nanofibers E2 and nanofibers E3, as shown in the red box, indicated that almost all the sharp peaks in the ATR‐FTIR spectra of FIN raw powders disappeared from the ATR‐FTIR spectra of nanofibers E2 and E3. These phenomena suggest the fine compatibility between EC and FIN, which can also be deduced from their molecular formula. FIN has –C═O groups in its molecules, which can form hydrogen bonds with –OH in EC molecules. Meanwhile, hydrophobic interaction can also form between them to promote their compatibility and the stability of co‐presence within the nanofibers E2 of the core sections of core–sheath nanofibers E3. Similarly, SDS molecules and PVP molecules have fine compatibility, as disclosed by the slightly blue box in Figure 8. Besides the hydrophobic interactions between PVP and SDS through carbon chains, electrostatic interactions can be formed between them for further compatibility and stability.

### 3.5 Physical properties of the electrospun nanofibrous membranes

**FIGURE 9:**
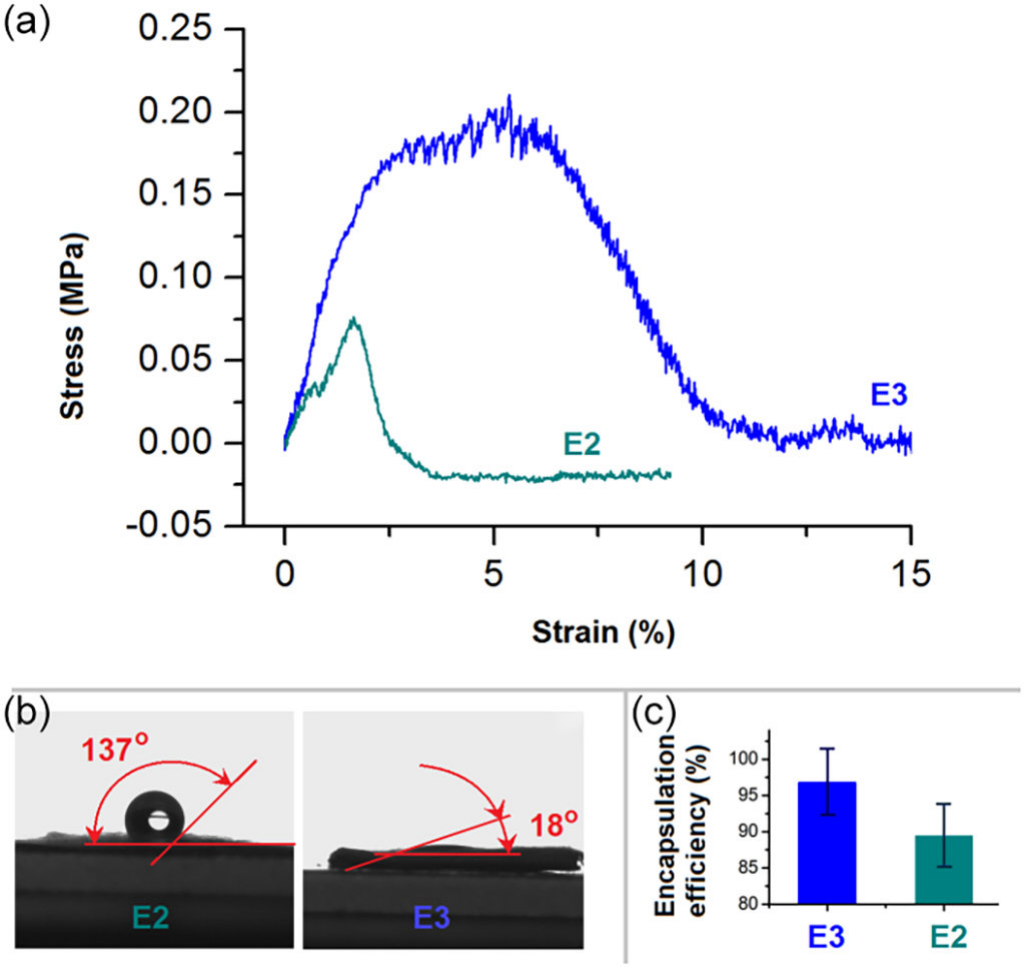
Comparisons between nanofibrous films E2 and E3: (a) typical stress–strain curves; (b) water contact angles; (c) FIN encapsulation efficiency.

Figure 9a presents the stress–strain profiles of the nanofibrous membranes E2 and E3. The nanofibrous membranes E2 have their TS and EI values of 5.43 ± 0.45 MPa and 70.67 ± 6.74%, respectively. In contrast, the nanofibrous membranes E3 have their TS and EI values of 5.43 ± 0.45 MPa and 70.67 ± 6.74%, respectively. The core–sheath nanofibers E3 show a significantly larger value of both TS and EI, suggesting a better mechanical performance than the blended nanofibers E2. These improvements should be attributed to the sheath PVP layer, which made the whole nanofibers more compact and stronger molecular entanglements.

Shown as Figure 9b, the nanofibrous membranes E2 and E3 have a WCA value of 122.40 ± 2.89° and 122.40 ± 2.89°, respectively. The core–sheath nanofibrous E3 exhibited a better hydrophilic property than the blended nanofibers E2. The reasons should be attributed to the property of the polymeric matrices, that is, EC of the blended nanofibers E2 and PVP of the surface of the core–sheath nanofibers E3. The better hydrophilicity of E3 is a positive factor for the transdermal drug delivery application.

### 3.6 The functional performances of the electrospun nanofibers

#### 3.6.1 Quantitative measurements of FIN

**FIGURE 10:**
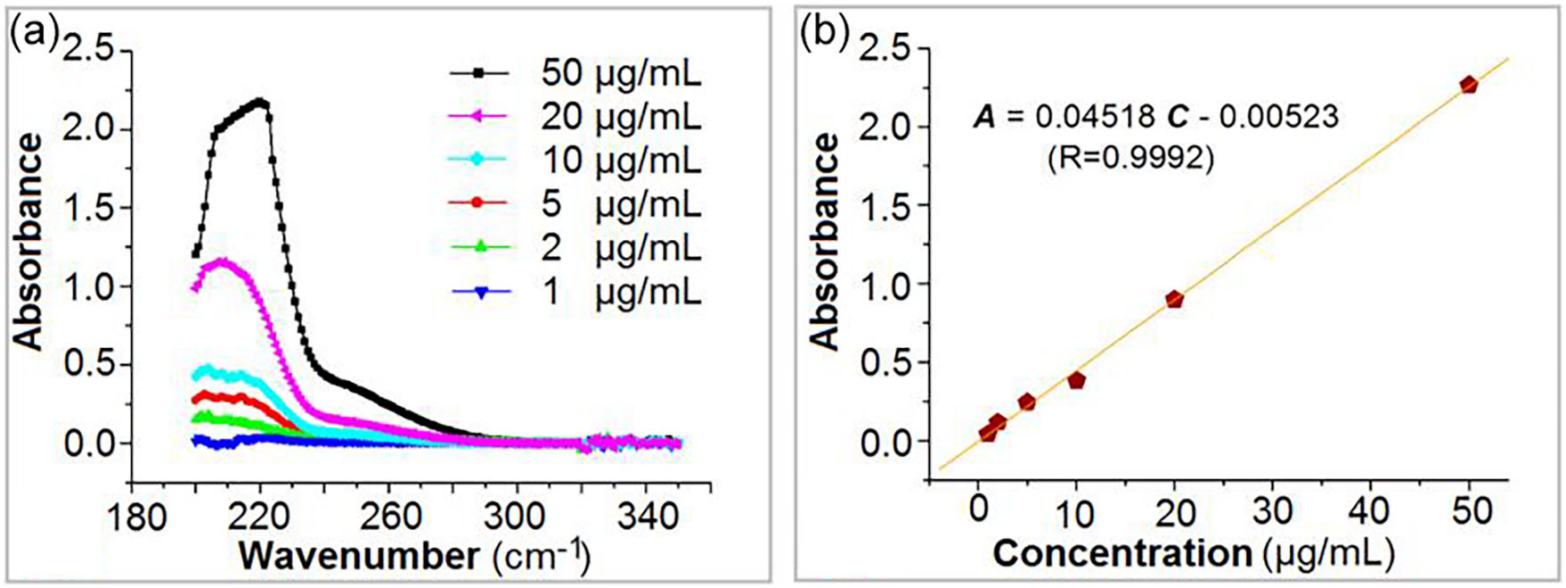
UV–Vis quantitative measurements of FIN: (a) the scanning lines of a series of standard solutions of FIN with a certain concentration; (b) the standard equation for measuring the concentration of FIN in the unknown solutions at *λ *= 220 nm.

FIN is a poorly water‐soluble drug. It has strong absorbances around 220 nm. The UV–Vis scanning lines of a series of standard solutions with a certain concentration of FIN are shown in Figure 10a. Based on the absorbance at the wavenumber of 220 nm, the standard equation for measuring the concentration of FIN in the unknown solutions can be regressed as *A* = 0.04518 C‐0.00523 (*R* = 0.9992) (Figure 10b).

#### 3.6.2 EE% and DL%

The measured EE% for nanofibers E2 and E3 were 89.5 ± 4.3% and 96.9 ± 4.6%, respectively. Apparently, the modified coaxial electrospinning provided a better performance in encapsulating the FIN molecules during the working process through the prevention of FIN molecules from the core FIN–EC fluids from reaching the environment by the sheath SDS–‐PVP fluids. This result concurred with other publications [76].

The measured DL% for nanofibers E2 and E3 were 10.7 ± 0.5% and 8.0 ± 0.4%, respectively. Although the core–sheath nanofibers E3 have a smaller DL% value than the monolithic nanofibers E2 due to the presence of sheath PVP and SDS, core–sheath nanostructures always show a better drug loading capability than the blended ones. During the preparations of nanofibers, the core sections of core–sheath nanofibers can even comprise pure drug molecules, that is, drug nano reservoirs or drug depots [?].

#### 3.6.3 FIN sustained release profiles from the monolithic nanofibers E2 and core–sheath nanofibers E3

**FIGURE 11:**
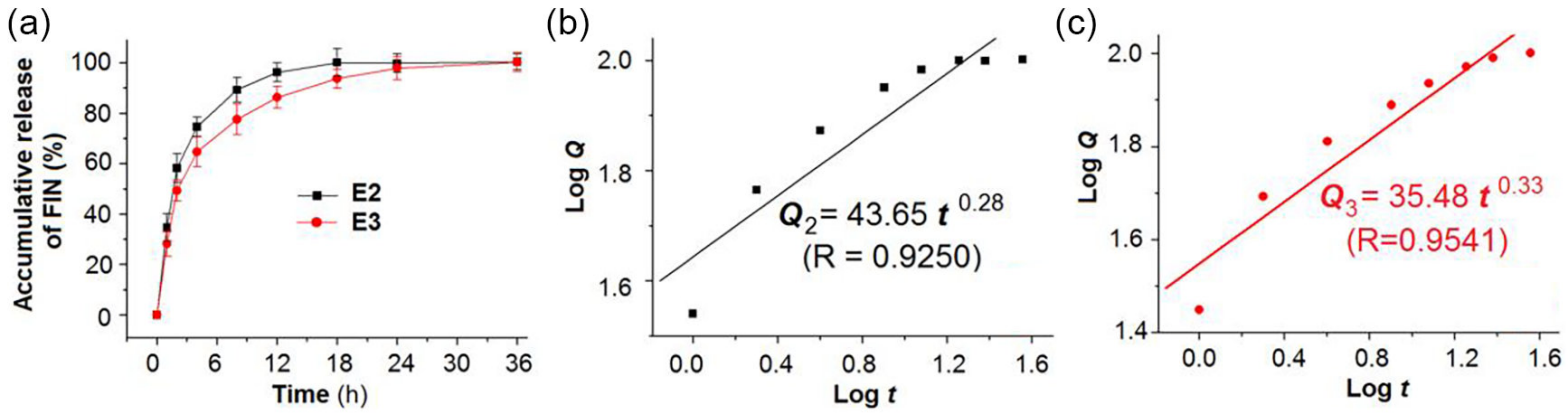
The in vitro drug release profiles of the nanofibers E2 and E3: (a) release profiles of FIN; (b) the regressed Peppas' equation from the drug release data of E2; and (c) the regressed Peppas' equation from the drug release data of E3.

The in vitro drug release profiles of the nanofibers E2 and E3 are included in Figure 11a. Both E2 and E3 were able to provide a typical drug‐sustained release profile over 24 h. To disclose the drug release mechanism, Peppas' equation was exploited to treat the in vitro release data. As shown in Figure 11b, the monolithic nanofibers E2 have an equation of *Q*_2_ = 43.65 *t* ^0.28^ (*R* = 0.9250). The value of *n* is 0.28, smaller than 0.45, giving a hint that the FIN molecules are released from the nanofibrous EC matrices through a typical Fickian mechanism [77]. Being an insoluble polymeric excipient, the drug molecules released from EC are always manipulated by the penetrations of water molecules from the bulk solution into the inner sections of the nanofibers, and later the dissolution of drug molecules and a reverse penetration of FIN molecules from the inner sections of nanofibers to the bulk solutions due to the concentration differences. As shown in Figure 11c, the core–sheath nanofibers E3 have an equation of *Q*_3_ = 35.48 *t* ^0.33^ (*R* = 0.9541). The value of *n* is 0.33, still smaller than 0.45, indicating that the FIN molecules are released from the nanofibrous core EC matrices through a typical Fickian mechanism. The sheath PVP section had a limited influence on the FIN release mechanism.

#### 3.6.4 FIN penetration performances from the nanofibers E2 and E3

**FIGURE 12:**
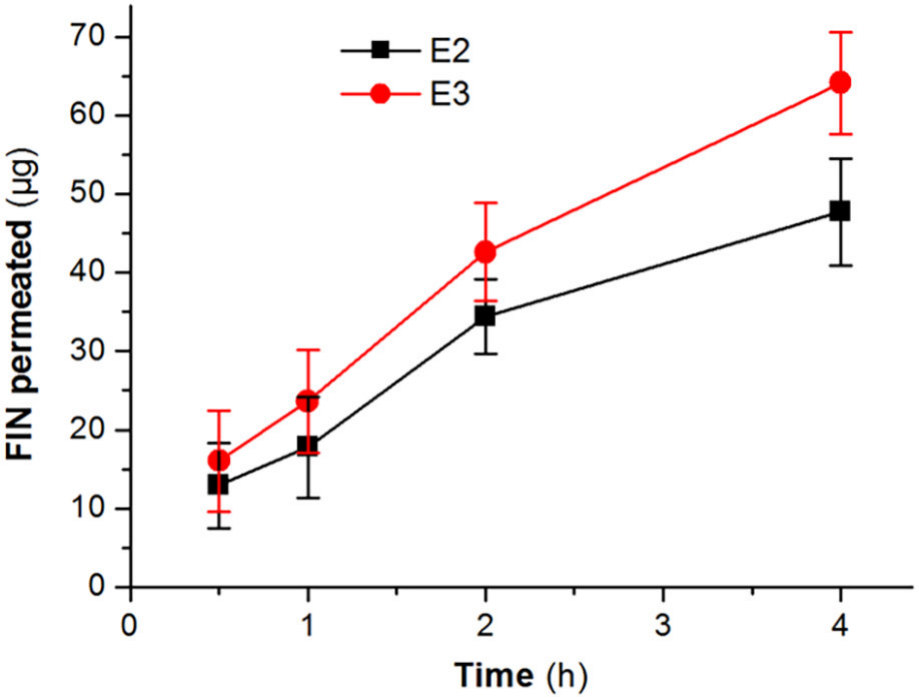
The ex vivo drug permeation performances.

The Ex vivo drug permeation performances of nanofibers E2 and E3 are included in Figure 12. Although the sustained release profiles of nanofibers E2 and E3 are similar, the penetration rates of FIN molecules from the nanofibers E2 and E3 always have significant differences from the initial tests at 0.5 h (10.32 ± 4.31% and 12.85 ± 5.14% for nanofibers E2 and E3, respectively) to the final tests at 4.0 h (38.16 ± 5.47% and 51.31 ± 5.19% for nanofibers E2 and E3, respectively). The permeation percentages of core–sheath nanofibers E3 were always higher than those of monolithic nanofibers E2. Apparently, the sheath sections containing PVP and SDS have played their roles in promoting a larger penetration section due to the following reasons. First, the SDS molecules are able to open the tight connections of the stratum corneum of the skin. SDS is insoluble in DCM and ethanol, and thus can’t be electrospun into the monolithic nanofibers E2 in a blended manner. Second, SDS molecules can be released all at once when the nanofibrous mats encounter the water for maximizing the initial “opening” effects owing to the easy dissolution of PVP K10. Third, PVP K10 molecules are able to promote the strong and close adhesion of the core EC‐FIN sections of E3 on the skin, facilitating the transportation of water molecules and drug molecules.

Regressed according to the linear equations, the FIN permeation amount (Q, µg) and time (t, h) have the relationships of *Q* = 10.0661t + 9.3262 (R = 0.9784, t < 4 h) and *Q* = 13.7513t + 10.8288 (*R* = 0.9911, t < 4 h) for the blended nanofibers E2 and the core–sheath nanofibers E3, respectively. Thus, the blended nanofibers E2 and the core–sheath nanofibers E3 had a permeability coefficient of 10.0661 and 13.8513 µg/h, respectively. The steady‐state fluxes for nanofibers E2 and E3 are 3.872 and 5.289 µg/h cm^2^, respectively. Compared with the blended nanofibers E2, the core–sheath nanofibers E3 can provide an increase in the permeation rate of (5.289–3.872)/3.872*100% = 36.6%.

The ex vivo permeation results indicate that the nanofibers E3 are able to provide a better potential transdermal drug delivery effect over the monolithic nanofibers E2. The most significant contributor should be the core–sheath nanostructures, by which the SDS molecules can be facilely co‐loaded into the nanofibers. What is more, the core–sheath nanostructures are able to provide a fast release of SDS and later a sustained release of the loaded FIN molecules for a maximized permeation performance. Based on the core–sheath nanofibers, a clear process‐structure‐performance relationship is thus clearly disclosed, which can be exploited for conceiving numerous other kinds of functional nanomaterials. Particularly, electrospinning has been demonstrated to create a wide variety of multi‐chamber nanostructures such as Janus [78, 79], tri‐chamber Janus [80, 81], tri‐chamber core–sheath [82, 83], and the integrated structures of Janus and core–sheath [84, 85, 86]. These advanced complex nanostructures can act as powerful platforms for supporting numerous novel nano drug delivery systems. Certainly, other popular techniques such as AI, Molecular Simulation [87], and new kinds of pharmaceutical excipients [76, 88, 89, 90] can also be integrated into these complex nanostructures for new possibilities. When conceiving novel nanofiber‐ and nanostructure‐based medicated nanomaterials, many excellent reviews and articles can be referred to for multifaceted paradigms [91, 92, 93, 94, 95, 96].

## 4 Conclusions

A modified coaxial electrospinning was successfully developed for creating transdermal drug delivery nanofibrous films of FIN in this study. The modified coaxial electrospinning was realized by a homemade concentric spinneret and the related homemade electrospinning apparatus. Although the single‐fluid blending electrospinning of EC‐FIN co‐dissolved solution was electrospinnable, the preparations of monolithic nanofibers E2 had poor robustness and contentiousness due to the easy clogging of the spinneret’s nozzle due to the high viscosity and easy evaporation of DCM and ethanol. The developed modified coaxial electrospinning, characterized by an unspinnable sheath fluid composed of PVP K10 and SDS, not only ensured a continuous and robust fabrication of nanofibers E3 but also endowed them with the core–sheath nanostructures, by which the transdermal enhancer SDS was co‐loaded. The micro‐formation mechanism about the influences of the unspinnable sheath solution on the creation of core–sheath nanofibers E3 is suggested. SEM tests verified that both the monolithic nanofibers E2 and core–sheath nanofibers E3 had a linear morphology without discerned beads or spindles, whereas the separate treatments of the sheath unspinnable solution containing PVP K10 and SDS resulted in microparticles E1. TEM evaluations demonstrated that both E1 and E2 were homogeneous polymeric composites, whereas nanofibers E3 had the obvious core–sheath nanostructures, with the core sections havping a larger density than the shell sections. The in vitro dissolution tests and ex vivo permeation tests suggested that the monolithic nanofibers E2 and the core–shell nanofibers E3 were able to provide a similar drug sustained release profile, whereas the latter could provide a better FIN permeation effect for potential transdermal drug delivery. Based on the protocols reported here, many other medicated nanofibers from the traditional single‐fluid blending electrospinning can be updated to endow them with a better functional performance or even a new therapeutic effect. Directly along this study, the following investigations are under research: 1) large‐scale productions of core–sheath nanofibers; 2) animal experiments and potential clinical applications; and 3) the co‐loading of phospholipid and drug molecules to further increase the penetration rate of FIN.

## Author Contributions

**Liang Sun** **:** data curation, formal analysis, investigation, validation, visualization, methodology, software, writing‐original draft. **Yisa Zhao and Xiyu Caomeng** **:** investigation, validation, methodology, writing‐review & editing. **Deng‐Guang Yu** **:** conceptualization, supervision, methodology, project administration, writing‐review & editing, funding acquisition. **Ping Liu** **:** conceptualization, supervision, project administration. All authors participated in discussions and manuscript preparations.

## Conflicts of Interest

The authors declare no conflicts of interest.

## Data Availability

Data will be made available on reasonable request to the corresponding authors.
